# The Bristol Medical School

**Published:** 1985-07

**Authors:** 


### Book Reviews

**THE BRISTOL MEDICAL SCHOOL**
**C. Bruce Perry**
**Published by**
**Bristol Branch of the Historical Association**
**Obtainable from**
**Department of History, University of Bristol**
**or**
**Peter Harris, 74 Bell Barn Road, Bristol 9**
**Pamphlet 90p.**

This pamphlet is basically the Creech Jones Memorial Lecture delivered by Professor Perry in the University of Bristol in 1983.

It is a factual and fascinating account of the history of the teaching of Medicine in Bristol from the day in 1737 when the Bristol Royal Infirmary opened its doors and the Apothecary – the resident general practitioner was allowed apprentices. The development of teaching is traced through the various schools that arose to foster it – the anatomy lectures at the Barber Surgeons Hall and the Red Lodge, the Bristol School of Anatomy and Medicine which provided dissecting rooms, the Lecture Courses at 3 College Green (now the nave of the Cathedral), the Bristol Medical and Surgical School at 3 King's Square, the union of these various institutions in 1834 to form the Bristol Medical School which in 1835 had 52 students and was the third largest provincial Medical School. We hear about the problems. In 1876 Examination results were so bad that there was a demand for the school to be inspected. The most intractable problem arose from the rivalry between the Royal Infirmary and the General Hospital. In 1919 the Medical Secretary of the Board of Education told a meeting of medical and university representatives in Bristol that it was 'all at sixes and sevens'. He urged the hospitals to unite. Mr. Henry Wills offered them £100,000 if they would do so. It took another 21 years and a world war before this was achieved.

These are the bare bones, but they are all well fleshed out with anecdote as those who know Professor Perry's style would expect. The great men whose lives and work have made the school are made to live – thus William Budd 'The idea that phthisis is a self propagating disease . . . disseminated by specific germs . . . first came to mind, unbidden so to speak, while I was walking on the Observatory Hill at Clifton in the second week in August in 1856'. Nice to think that the incomparable views from there remain unspoiled, that Brunel's bridge which Budd would have seen unfinished and abandoned is complete and still perfect and that it is still a place to go and ponder a diagnostic problem.

Any member of the Bristol Medical School who wants to know more about his professional family history will find this pamphlet is what he is looking for.

